# Novel equation to determine the hepatic triglyceride concentration in humans by MRI: diagnosis and monitoring of NAFLD in obese patients before and after bariatric surgery

**DOI:** 10.1186/s12916-014-0137-y

**Published:** 2014-08-26

**Authors:** Raúl Jiménez-Agüero, José I Emparanza, Adolfo Beguiristain, Luis Bujanda, José M Alustiza, Elisabeth García, Elizabeth Hijona, Lander Gallego, Javier Sánchez-González, María J Perugorria, José I Asensio, Santiago Larburu, Maddi Garmendia, Mikel Larzabal, María P Portillo, Leixuri Aguirre, Jesús M Banales

**Affiliations:** Department of Liver and Gastrointestinal Diseases, Biodonostia Research Institute, Donostia University Hospital (HUD), University of the Basque Country (UPV/EHU), San Sebastian, Spain; Clinical Epidemiology Unit, CASPe, CIBER-ESP, Biodonostia Research Institute – HUD, San Sebastian, Spain; National Institute for the Study of Liver and Gastrointestinal Diseases (CIBERehd), Barcelona, Spain; Osatek SA, San Sebastian, Spain; Philips Healthcare, Iberia, Madrid Spain; IKERBASQUE, Basque Foundation for Science, Bilbao, Spain; Department of Pathology, HUD, San Sebastian, Spain; Department of Nutrition and Food Science, Faculty of Pharmacy, UPV/EHU, CIBER-obn, Vitoria, Spain

**Keywords:** Non-alcoholic fatty liver disease, Magnetic resonance imaging, Hepatic fat concentration, Obesity, Bariatric surgery, Diagnosis

## Abstract

**Background:**

Non-alcoholic fatty liver disease (NAFLD) is caused by abnormal accumulation of lipids within liver cells. Its prevalence is increasing in developed countries in association with obesity, and it represents a risk factor for non-alcoholic steatohepatitis (NASH), cirrhosis and hepatocellular carcinoma. Since NAFLD is usually asymptomatic at diagnosis, new non-invasive approaches are needed to determine the hepatic lipid content in terms of diagnosis, treatment and control of disease progression. Here, we investigated the potential of magnetic resonance imaging (MRI) to quantitate and monitor the hepatic triglyceride concentration in humans.

**Methods:**

A prospective study of diagnostic accuracy was conducted among 129 consecutive adult patients (97 obesity and 32 non-obese) to compare multi-echo MRI fat fraction, grade of steatosis estimated by histopathology, and biochemical measurement of hepatic triglyceride concentration (that is, Folch value).

**Results:**

MRI fat fraction positively correlates with the grade of steatosis estimated on a 0 to 3 scale by histopathology. However, this correlation value was stronger when MRI fat fraction was linked to the Folch value, resulting in a novel equation to predict the hepatic triglyceride concentration (mg of triglycerides/g of liver tissue = 5.082 + (432.104 * multi-echo MRI fat fraction)). Validation of this formula in 31 additional patients (24 obese and 7 controls) resulted in robust correlation between the measured and estimated Folch values. Multivariate analysis showed that none of the variables investigated improves the Folch prediction capacity of the equation. Obese patients show increased steatosis compared to controls using MRI fat fraction and Folch value. Bariatric surgery improved MRI fat fraction values and the Folch value estimated in obese patients one year after surgery.

**Conclusions:**

Multi-echo MRI is an accurate approach to determine the hepatic lipid concentration by using our novel equation, representing an economic non-invasive method to diagnose and monitor steatosis in humans.

## Background

Non-alcoholic fatty liver disease (NAFLD) is triggered by intra-hepatocellular accumulation of lipids (mainly triglycerides) and affects up to 30% of the Western population [1,2]. Its pathogenesis usually involves the so-called metabolic syndrome linked to obesity, diabetes, hypertension, hypertriglyceridemia and/or insulin resistance. Although NAFLD generally presents a benign course, it may progress to non-alcoholic steatohepatitis (NASH) and to the development of cirrhosis and hepatocellular carcinoma [2].

NAFLD is usually asymptomatic at diagnosis [3]. Therefore, determination of the hepatic lipid content poses a major challenge in terms of identification, treatment and control of the disease progression [4]. Currently, the standard procedure used to evaluate hepatic steatosis is the histopathological examination of cross-liver sections and the semi-quantitative estimation of the percentage of hepatocytes (0% to 100%) containing macrovesicular fat, which is graded on a 0 to 3 scale [5]. However, this is an expensive and invasive practice that presents inherent risks. Moreover, it only provides a two-dimensional estimation of a particular biopsy and is subject to inter-individual visual evaluation depending on the pathologist’s training, which usually results in overestimation of the liver fat content [6]. On the other hand, evaluation of steatosis using computational image analysis of histology slides is possible [7,8], but is not carried out routinely in clinical practice. Therefore, it is essential to establish new non-invasive approaches to accurately determine the hepatic fat concentration, allowing the correct diagnosis and monitoring of steatosis.

Magnetic resonance imaging (MRI) represents a potential non-invasive technique for assessing hepatic steatosis in three-dimensions [9]. It measures the proportion of the mobile proton density of the liver that is attributable to fat [10]. Increasing evidence, using fat-water phantoms and/or histopathological semi-quantitative analysis of hepatic fat as reference standards, suggests that MRI may represent an accurate method to determine the hepatic lipid content [10,11]. However, additional research is still needed to validate this hypothesis and, more importantly, to test the potential of MRI as a tool to determine the hepatic triglyceride concentration (that is, Folch value).

The purpose of our prospective study of diagnostic accuracy was to evaluate the potential of multi-echo MRI to quantitate the hepatic triglyceride concentration. Multi-echo MRI fat fractions were compared with the hepatic steatosis determined by either histopathology or biochemical methods in patients operated for morbid obesity or who underwent liver surgery. Moreover, we analyzed the role of multi-echo MRI to monitor steatosis in morbidly obese patients after bariatric surgery. Our results indicate that multi-echo MRI is a precise method to determine the hepatic triglyceride concentration utilizing a novel equation and may be routinely employed in clinical practice to diagnose and monitor steatosis.

## Methods

### Patient selection and study design

We designed a prospective and cross-sectional single-site study of diagnostic accuracy (from January 2009 to January 2014), which was approved by the Ethics Committee of the Donostia University Hospital as defined by Spanish law and European directives. An informed consent form approved by the institutional review board was signed by all patients before starting the study.

For the non-obese group, we included all consecutive patients referred to our hospital during 2009 for hepatic surgery due to different etiologies without underlying liver disease. The inclusion criterion was a body mass index (BMI) <35 kilograms per square meter.

On the other hand, we included all consecutive obese patients referred to our hospital between 2010 and 2013 for bariatric surgery (gastric sleeve or gastric bypass) or for partial liver resection due to metastatic liver disease without underlying hepatic disease. The inclusion criterion for obese patients was BMI ≥35 kilograms per square meter. All the patients in this group ranged from 36 to 63 BMI and were classified as obesity class II (BMI = 35 to 39.9) or III (BMI ≥40) by the world health organization (WHO) [12]. All obese patients undergoing bariatric surgery had surgical indication for BMI ≥40 or BMI = 35 to 39.9 with significant morbidity [12].

Multi-echo MRI was performed in all patients under study the day before surgery, and a liver biopsy was obtained at the same time as the bariatric or hepatic surgery. Importantly, the MRI-biopsy period was less than 24 hours. Liver biopsies were processed for histopathological studies as well as for quantification of the hepatic fat concentration measured by a lipid assay (see below). Expert professionals involved in each quantification method (that is, MRI, histopathology and lipid assay) performed the analysis blindly.

A validation group with 31 additional patients (24 obese and 7 non-obese) was also included between June 2013 and January 2014 to confirm the efficiency of our new formula to predict the hepatic Folch value from multi-echo MRI. Thus, multi-echo MRI before surgery as well as liver biopsy at the moment of operation were obtained from these patients to compare the measured (by Folch) and the predicted (by MRI) liver triglyceride concentration.

Finally, the aforementioned obese patients received a second multi-echo MRI one year after surgery in order to quantitate and monitor steatosis. In addition, a multivariate analysis (initial Folch estimated, total weight, weight loss, age and gender) of multi-echo MRI fat fraction and Folch estimated was performed.

### Clinical data

Gender and age were considered in all patients. Body measurements included weight and standing height at the moment of the liver biopsy. The BMI was calculated from these values. The presence of associated diseases such as diabetes, hyperlipidemia and obstructive sleep apnea was checked in all patients. In addition, treatment with drugs (that is, contraceptives, statins, immunosuppressives, antidepressants, nifedipines, hormones and paracetamol) was also considered. Biological data for each patient included hematological parameters, liver function tests and lipid profiles.

### Liver histology

Liver biopsies were double-blind examined by two expert liver pathologists. All biopsies were obtained from a hepatic wedge in the left anterior liver. Tissue samples were fixed in 10% formalin solution and embedded in paraffin. Sections 4 μm thick were routinely stained with hematoxylin-eosin. Furthermore, liver steatosis was reported as a semi-quantitative evaluation of the percentage of hepatocytes (0% to 100%) containing macrovesicular fat (that is, lipid droplets equal to or larger than the size of the nucleus, often displacing the nucleus) or microvesicular fat (that is, numerous small fat droplets surrounding a centrally located nucleus). Results were expressed in terms of fat percentage in hepatocytes and ranged from 0 to 3 (that is, 0: no fat, 1: up to 33% fat, 2: 33% to 66% fat, and 3: >66% fat) [13].

### Hepatic lipid assay

The lipid concentration of the liver was determined according to the method described by Folch *et al.* [14]. This biochemical approach determines the triglyceride concentration in liver samples (mg of triglyceride/g of liver tissue) and was used as a gold standard (that is, reference method) to compare with both MRI data and histology. This method, with some minor modifications, continues to be considered the classic and most reliable approach for quantitatively extracting lipids [15]. Two experienced researchers performed the Folch determinations from liver biopsies without knowing any clinical data or MRI/histological results.

Briefly, liver tissue was washed with saline solution to eliminate any traces of blood and subsequently homogenized with 2:1 chloroform/methanol solution. Samples were then incubated at 50°C for 30 minutes and with 2 ml of KCl 0.1 M to speed up the phase separation process; this mixture was shaken for one minute. Samples were kept for two hours at 4°C, and then centrifuged at 2,000 to 3,000 rpm for 20 minutes to facilitate the separation of the upper phase (or aqueous methanol dragging) and the lower phase (or chloroform phase) containing the lipids. Most of the aqueous phase was removed and the chloroform phase adjusted to a known final volume with chloroform. A volume of 1 ml of the chloroform phase was transferred into a tube previously weighed and the solution was evaporated by drying using a nitrogen stream. The tube was weighed again and the amount of fat calculated by the gravimetric method. Finally, lipids were dissolved in isopropanol and triglycerides measured by spectrophotometry using a commercial kit from Spinreact (SantEsteve de Bas, Spain).

### Multi-echo magnetic resonance imaging

The multi-echo MRI technique to assess the tissue fat content was performed as we have previously reported in animal models [16]. Briefly, this method is based on a three-dimensional multi-echo gradient sequence acquired in axial orientation with 12 different echoes (TE min = 1.04 ms, δTE = 0.78 ms, TE Final = 25.14 ms, TR = 72 ms, Flip Angle = 25°, FOV 375/328 mm, matrix resolution 232/129). Images were achieved for spectral analysis of the MRI signal to distinguish between fat and water content in each image pixel. The three-dimensional acquisition (10 consecutive slices: slice thickness = 12 mm) was performed in a single breath-hold of 20 seconds that resulted in a final image of the whole liver anatomy. All the acquisitions were carried out in a 1.5 T Achieva System (Philips Healthcare, Best, The Netherlands). An integrated quadrature body coil was used in obese patients to fit better inside the scanner. For non-obese patients, images were acquired using a 16-channel phased array coil maintaining the same image parameters previously described in the acquisition method.

Quantitative analysis of the images was performed following a previously published methodology [16]. This approach was implemented in an in-line PRIDE tool that runs in a MR Work Station (Extended Work Space, Philips Healthcare). Importantly, native multi-echo images were not directly analyzed by the radiologist. The software automatically generates the water and fat intensity maps, the water and fat R2* (reciprocal of T2*) maps, and fat fraction maps. Water and fat signal maps are then analyzed by the radiologist as a conventional parametric map (region of interest (ROI) analysis) to calculate the final fat fraction measured as a percentage. The analysis of three ROIs was performed in the fat fraction maps manually dressed in segment III, near to the lower border of the liver, avoiding vascular vessels. The average of three measured fat fraction values was calculated for each patient to provide the final fat fraction content. The radiologist was not aware of other test results. Therefore, the data were not subject to any bias.

### Statistical analysis

Quantitative variables were expressed as mean ± standard deviation and categorical variables as absolute and relative frequencies. Coefficient of variation was estimated as a measure of dispersion of continuous data. We used the Student *t* test for statistical comparisons between two groups of normally distributed variables and Mann–Whitney tests for non-parametric methods. The association of variables with the lipid content in liver tissue (Folch and histology) was carried out by linear regression analysis and Pearson correlation coefficient (r) in the case of continuous variables, and by one-way analysis of variance (ANOVA) for categorical variables (or non-parametric tests when necessary).

We considered the Folch determination as the reference test of the liver fat content. To measure the ability of MRI to provide a quantitative liver fat content, we used a linear regression model. All the variables showing a relation with the Folch determination in the univariate analyses with a *P* value lower than 0.20 were selected for a multivariate model. Using a backwards stepwise linear model we estimated their ability to contribute to improve Folch prediction over the model with MRI alone.

We constructed a calibration graph for the validation dataset and studied the relation between estimated and measured Folch value using a linear regression model.

To assess the effect of clinical parameters on liver fat improvement as measured by MRI (difference between initial MRI-estimated and final MRI-estimated liver fat content), we used backwards stepwise linear regression.

Results were considered to be statistically significant at *P* <0.05. Statistical analysis was performed using STATA® SE v13 software (StataCorp. LP, Drive College Station, TX USA).

## Results

### Clinical and biological human data

One hundred and twenty nine adult patients (52 men and 77 women) with a mean age of 50 years (50.2 ± 14.3) were included in the study between January 2009 and January 2014 (Table 1). Patients were clustered in two groups: 1) a control group that underwent liver surgery (n = 32), and 2) a group of obese patients (n = 97). Patients in the control group underwent partial liver resection for different etiologies that justified the intervention during 2009, that is, 22 metastases, 3 hepatocarcinomas, 1 cholangiocarcinoma, 3 adenomas and 3 hemangiomas. Among the obese patients, 86 underwent bariatric surgery and 11 partial liver resection between 2010 and 2013.

**Table 1:** **Clinical features of the population under study**

**Clinical characteristics**	**Control group (number = 32)**	**Obese group (number = 97)**	***P*** **value**	**Overall (number = 129)**
Age (years)	60.9 ± 11.5	46.7 ± 13.4	<0.0001	50.2 ± 14.3
Weight (Kg)	71.9 ± 13.6	120.7 ± 26.3	<0.0001	108.7 ± 31.8
BMI (Kg/m^2^)	26 ± 4.5	44.6 ± 7.9	<0.0001	40 ± 10.8
**Associated diseases and treatments**				
Diabetes	5 (15.63%)	28 (28.86%)	N.S.	33 (25.58%)
Dyslipidemia	10 (31.25%)	36 (37.11%)	0.054	46 (35.66%)
Obstructive sleep apnea	1 (3.1%)	32 (33%)	<0.001	33 (25.6%)
Contraceptive	1 (3.1%)	3 (3.1%)	N.S.	4 (3.1%)
Statin	3 (9.3%)	17 (17.5%)	N.S.	20 (15.5%)
Immunesuppresive	1 (3.1%)	5 (5.15%)	N.S.	6 (4.65%)
Antidepressant	2 (6.2%)	11(11.34%)	N.S.	13 (10.07%)
Nifedipine	0 (0%)	10 (10.3%)	N.S.	10 (7.75%)
Hormone	1 (3.1%)	6 (6.18%)	N.S.	7 (5.42%)
Paracetamol	2 (6.2%)	7 (7.21%)	N.S.	9 (6.97%)
**Serological assessments**				
AST (U/L)	26.8 ± 12.9	24.7 ± 12.9	N.S.	25.2 ± 12.9
ALT (U/L)	31.8 ± 20	32.7 ± 21.8	N.S.	32.4 ± 21.3
GGT (U/L)	53 ± 46.4	35.6 ± 29.3	N.S.	40.2 ± 35.3
ALP (U/L)	99.9 ± 48.5	71.6 ± 22.4	<0.01	79.7 ± 34.3
Triglycerides (mg/dL)	106.9 ± 54.1	163.3 ± 154.8	<0.01	148.5 ± 137.8
Cholesterol (mg/dL)	190.6 ± 54.1	200.8 ± 42.6	N.S.	198.2 ± 45.9
**Liver triglyceride content – Biochemical assay (mg/g)**	33.3 ± 28.3	94.5 ± 57.1	<0.0001	79.3 ± 57.8

ALP, alkaline phosphatase; ALT, alanine aminotransferase; AST, aspartate aminotransferase; BMI, body mass index; GGT, gamma glutamyl transpeptidase.

The group of obese patients had a mean age (46.7 ± 13.4 years) that was lower than that of the control group (60.9 ± 11.5 years) (*P* <0.0001), since obesity was observed in middle-aged persons, and the control group involved liver surgery in older patients. On the other hand, as expected, the group of obese patients showed increased body weight (120.7 ± 26.3 *versus* 71.9 ± 13.6 Kg) and BMI (44.6 ± 7.9 *versus* 26 ± 4.5 Kg/m^2^) compared to the control group (*P* <0.0001 in both comparisons), as well as increased obstructive sleep apnea (33% *versus* 3.1%; *P* <0.001), serum triglycerides (163.3 ± 154.8 *versus* 106.9 ± 54.1 mg/dL; *P* <0.01) and hepatic triglyceride content (94.5 ± 57.1 versus 33.3 ± 28.3 mg/g; *P* <0.0001). Finally, the serological levels of alkaline phosphatase (ALP) were decreased in the obese patients compared to controls (71.6 ± 22.4 *versus* 99.9 ± 48.5 U/L; *P* <0.01).

### Obese patients showed increased hepatic steatosis compared to controls by histopathological examination

**Figure 1:**
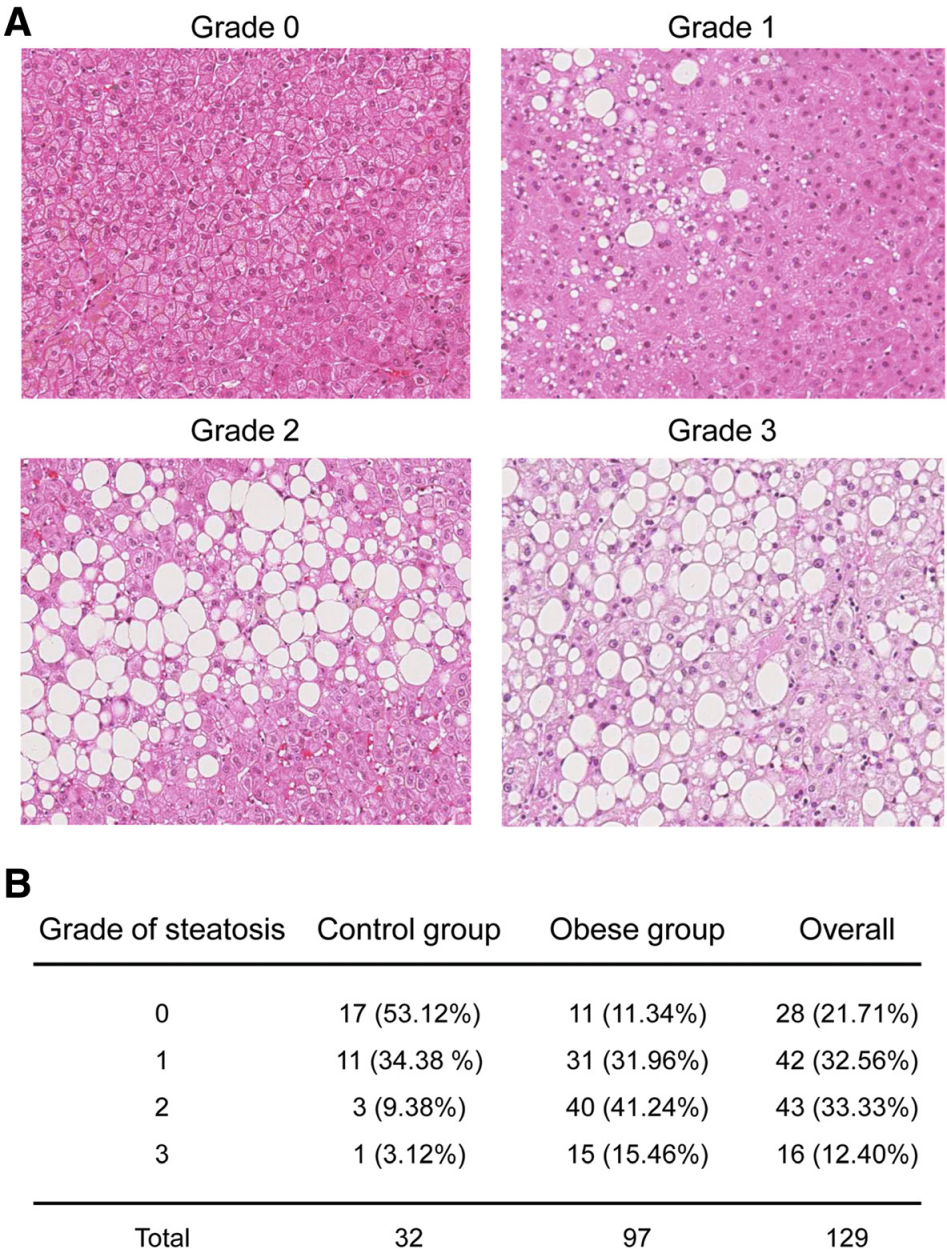
**Histopathological examination of steatosis in obese and control patients. A)** Representative images with hematoxylin-eosin staining from patients with different degrees of liver steatosis (grades 0 to 3). **B)** Obese patients showed increased degrees of steatosis compared to non-obese patients who underwent liver surgery. The overall prevalence of hepatic steatosis in our study was 78.29% (101 of 129 patients had macrovesicular steatosis ≥1 at histopathological analysis).

The analysis of steatosis by histopathology showed different degrees of hepatic fat content between the obese and control group (Figure 1). A total of 53.12% of patients in the control group had no hepatic fat, 34.38% showed mild, 9.38% moderate and 3.12% severe macrovesicular steatosis. On the other hand, 11.34% of patients with obesity had no hepatic fat, 31.96% showed mild, 41.24% moderate and 15.46% severe macrovesicular steatosis. Overall, the prevalence of hepatic steatosis in this study population was 78.29% (101 of 129 patients had macrovesicular steatosis ≥1 at histopathological analysis). Importantly, no adverse events associated with the liver biopsy collection were reported in any patient.

### Correlation analysis between multi-echo MRI fat fraction and histopathological estimation of hepatic steatosis in humans

**Figure 2:**
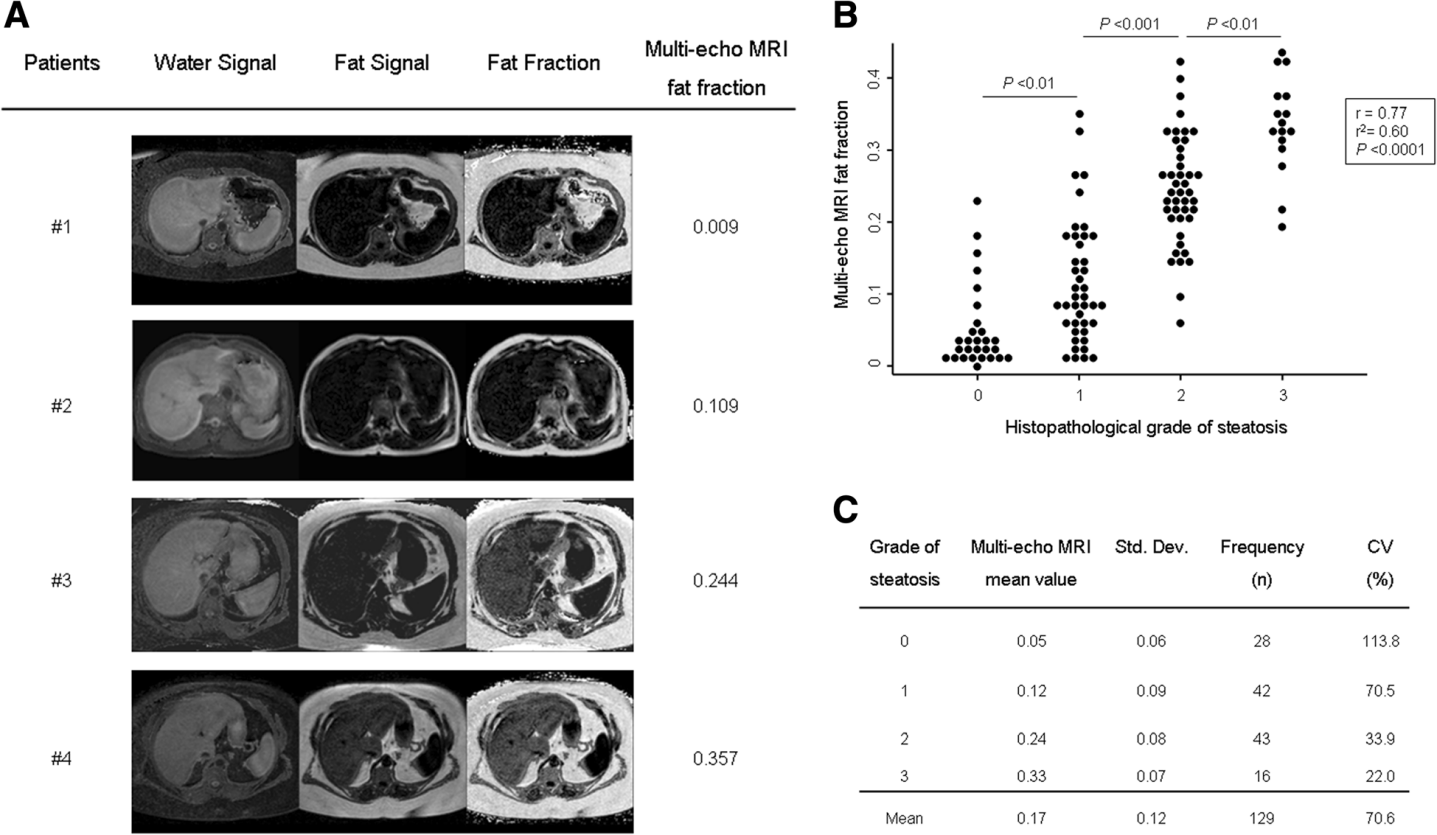
**Multi-echo MRI fat fractions positively correlate with the grade of steatosis estimated by histopathological measurements. A)** Representative multi-echo MRI images showing different degrees of water and fat intensity, and fat fraction in different patients. **B)** Multi-echo MRI fat fractions positively correlate with the grade of steatosis estimated by histopathological measurements in human livers (n =129). Dots represent the values of each case. **C)** Multi-echo MRI fat fraction mean values of each steatosis grading group (0 to 3 scale). CV, coefficient of variation; MRI, magnetic resonance imaging; Std. Dev, standard deviation.

In all 129 patients under study, we evaluated the potential correlation between the multi-echo MRI fat fraction and the semi-quantitative analysis of liver steatosis by histopathological measurement (Figure 2). Our data showed that multi-echo MRI fat fractions positively correlate with the grade of steatosis estimated by histopathological measurements (r = 0.77, r^2^ = 0.60; *P* <0.0001). However, importantly, although significant differences in the multi-echo MRI fat fraction values were found between all the groups of patients graded on a 0 to 3 scale of steatosis, there was intragroup variability in the MRI scores (mean coefficient of variation (CV) = 70.6%, Figure 2). Importantly, no adverse events associated with multi-echo MRI were reported in any patient.

### Correlation analysis between the histopathological estimation of hepatic steatosis in humans and the biochemical measurement of the hepatic lipid concentration

**Figure 3:**
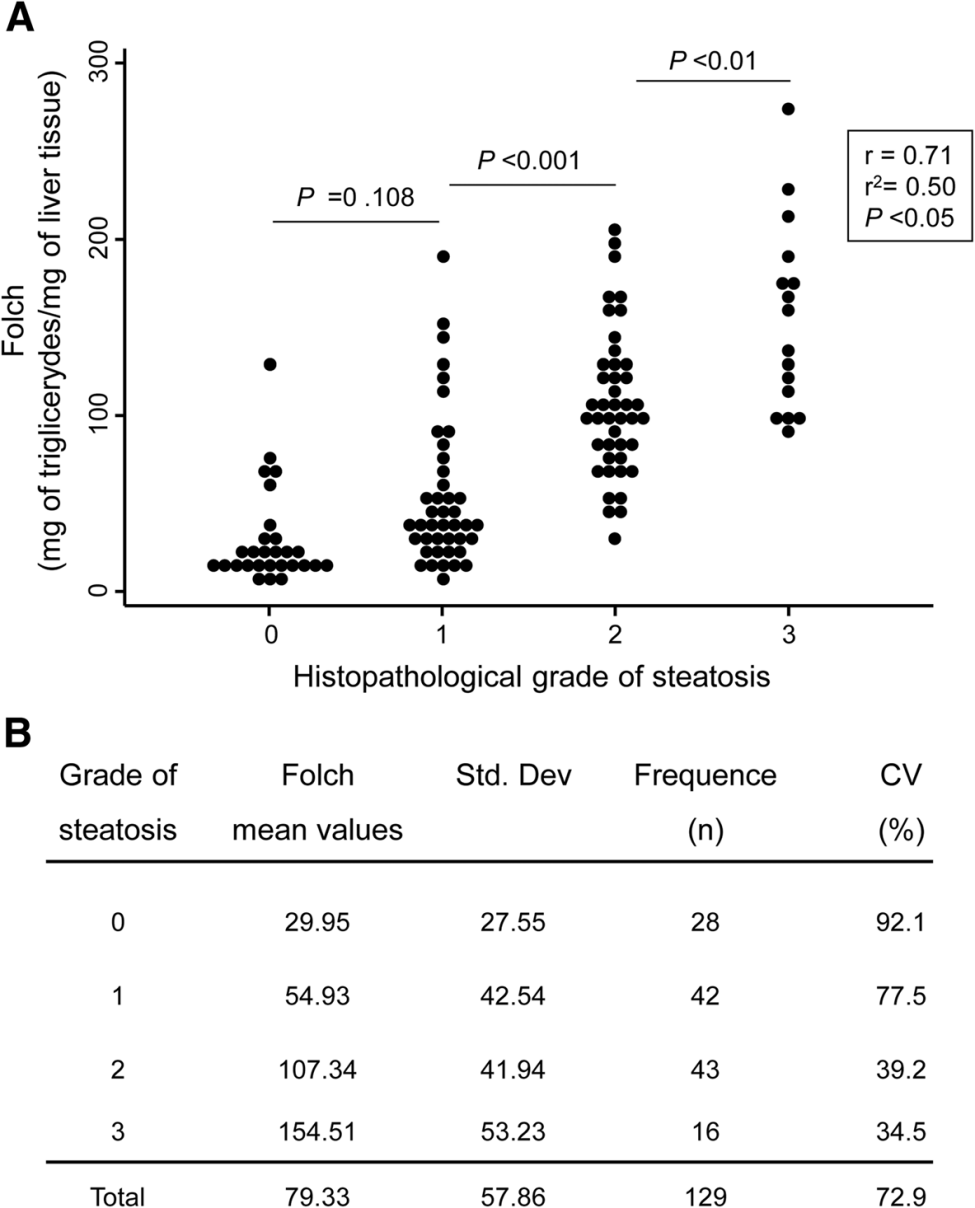
**The hepatic triglyceride concentration (Folch) positively correlates with the grade of steatosis estimated by histopathological measurements. A)** Correlation between Folch values and the grade of steatosis estimated by histopathological measurements in human livers (n =129). Dots represent the values of each case. **B)** Folch mean values of each steatosis grading group (0 to 3 scale). CV, coefficient of variation; Std. Dev, standard deviation.

We studied the potential correlation between the semi-quantitative analysis of steatosis in human liver biopsies by histopathology and the direct quantification of the hepatic lipid concentration (that is, Folch value) (Figure 3). Our data indicated that Folch values positively correlate with the grade of steatosis estimated by histopathological measurements (r = 0.71, r^2^ = 0.50; *P* <0.05). In this regard, significant differences in the Folch values were found between histological grades 1, 2 and 3, but not between grades 0 and 1. Moreover, importantly, there was intragroup Folch variability in all four groups of steatosis determined histopathologically on a 0 to 3 scale (mean CV = 72.9%, Figure 3).

### Correlation analysis between multi-echo MRI fat fraction and the biochemical measurement of the hepatic lipid concentration

**Figure 4:**
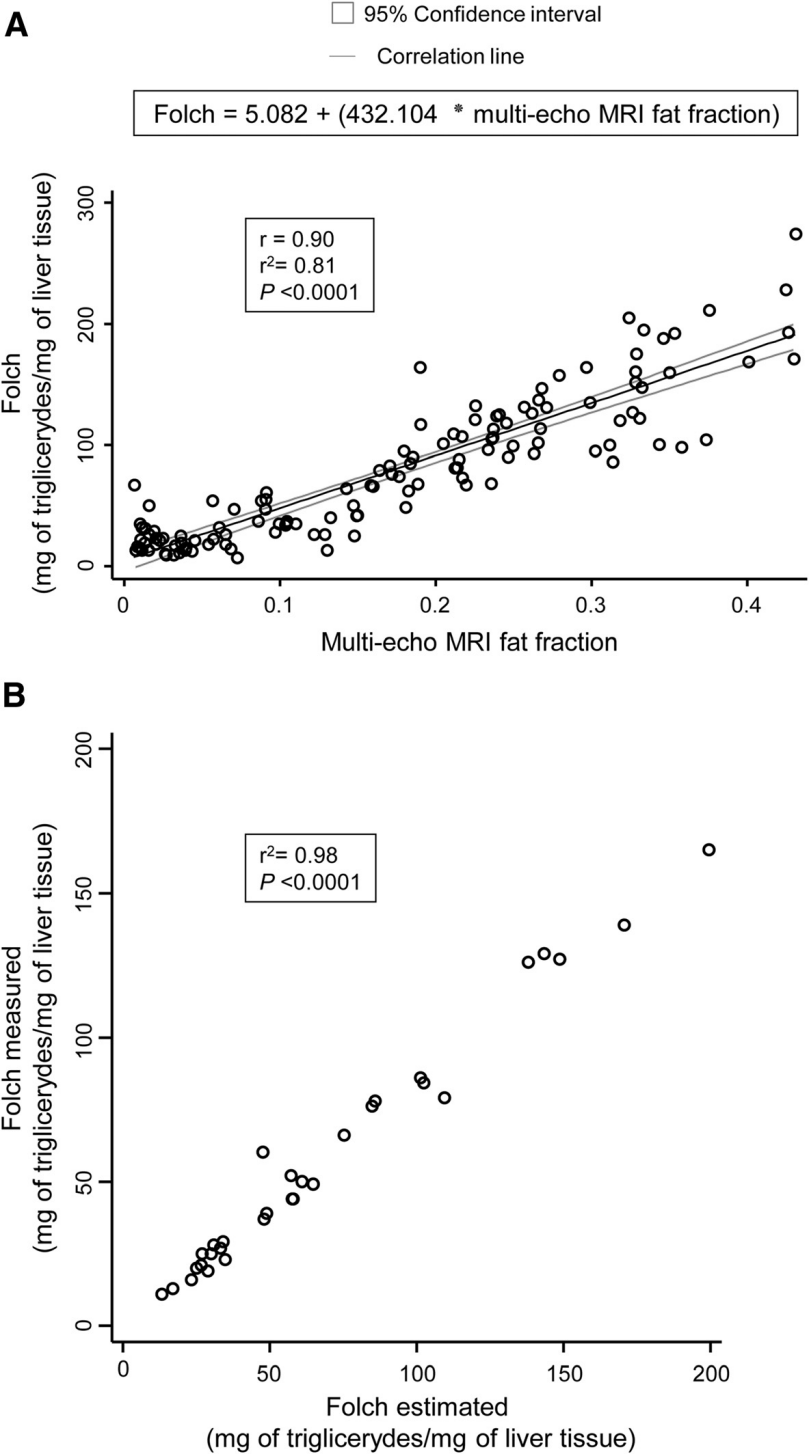
**Multi-echo MRI fat fractions positively correlate with the hepatic triglyceride concentration. A)** Correlation between the Folch values and the multi-echo MRI fat fraction in human livers (n =129). Box includes the resultant equation that predicts the Folch values from multi-echo MRI fat fractions. **B)** Calibration plot between the measured and estimated Folch values using the prediction formula [Folch = 5.082 + (432.104 * multi-echo MRI fat fraction)]. Dots represent the values of each case. MRI, magnetic resonance imaging.

Next, we evaluated the potential correlation between the multi-echo MRI fat fraction of human livers and the direct quantitation of steatosis through the biochemical measurement of the hepatic triglyceride concentration (Folch value) (Figure 4A). Our results indicated that the multi-echo MRI fat fractions positively correlate with the Folch values (r =0.90, adjusted r^2^ = 0.81; *P* <0.0001) resulting in the following equation:

$$ \mathrm{Folch}\ \left(\mathrm{mg}\ \mathrm{of}\ \mathrm{triglycerides}/\mathrm{g}\ \mathrm{of}\ \mathrm{liver}\right) = 5.082+\left(432.104*\mathrm{multi}-\mathrm{echo}\ \mathrm{MRI}\ \mathrm{fat}\ \mathrm{fraction}\right) $$

### Calculation of the hepatic fat concentration through the multi-echo MRI fat fraction

The aforementioned equation was evaluated with a new validation group of patients. Thus, 31 additional adult patients (24 obese and 7 controls) underwent multi-echo MRI, and their fat fraction was employed to predict their hepatic fat concentration (Folch) by using the formula. Next, these predicted Folch values were compared with the direct biochemical measurement of the hepatic fat concentration. Our data showed that the equation is able to predict the hepatic triglyceride concentration with a strong correlation between 1) the biochemically measured Folch values and 2) the estimated Folch values obtained with the formula (r^2^ = 0.98; *P* <0.0001) (Figure 4B).

### Univariate analyses of hepatic fat concentration and multivariate evaluation of multi-echo MRI prediction

We tested the role of the different variables analyzed with the Folch method (Tables 2 and 3). The univariate studies showed that Folch values are lower in male than female patients, and higher in patients with apnea (*P* <0.05 and *P* <0.0001, respectively; Table 2). In addition, Folch values correlate negatively with age (*P* <0.05; Table 3), and positively with BMI (*P* <0.0001; Table 3), alanine aminotransferase (ALT), triglycerides and cholesterol (*P* <0.05 for all three comparisons; Table 3). On the other hand, the multivariate analysis indicated that these variables do not improve the Folch predictive capacity obtained by the aforementioned formula (data not shown).

**Table 2:** **Univariate analysis of categorical variables in relation to the measured Folch values**

**Categorical variables**	**Type or presence/absence**	**Number**	**Folch**	**Std. Dev.**	***P*** **value**
Gender	Men	52	66.98	50.99	<0.05
	Women	77	87.68	60.98	
Diabetes	Yes	33	89.36	59.02	N.S.
	No	96	75.87	57.36	
Dislipemia	Yes	46	92.61	61.12	0.05
	No	83	71.96	54.97	
Contraceptive	Yes	4	59.75	38.11	N.S.
	No	124	79.78	58.58	
Statin	Yes	20	102.23	87	N.S.
	No	108	74.88	56.55	
Immunosuppressive	Yes	5	117.5	75.00	N.S.
	No	123	77.6	56.12	
Antidepressant	Yes	12	113.02	85.78	N.S.
	No	116	75.65	53.72	
Nifedipine	Yes	10	92.82	62.59	N.S.
	No	118	78	57.79	
Hormones	Yes	7	86.71	60.62	N.S.
	No	121	78.72	58.13	
Paracetamol	Yes	9	91.55	78.82	N.S.
	No	119	78.22	56.51	
Apnea	Yes	33	116.48	59.4	<0.0001
	No	96	66.55	51.75	

Std. Dev., standard deviation.

**Table 3:** **Summary of the univariate regression models between continuous variables and the measured Folch values**

**Continuous variables**	**Constant**	**Coefficient**	**r** ^**2**^	***P*** **value**
Age	122.28	−0.85	0.04	<0.05
Weight	−4.59	0.77	0.18	<0.0001
Height	200.65	−0.74	0.01	N.S.
BMI	−27.24	2.66	0.25	<0.0001
AST	67.79	0.44	0.01	N.S.
ALT	60.33	0.57	0.04	<0.05
GGT	84.74	−0.15	0.01	N.S.
ALP	92.32	−0.21	0.02	N.S.
Triglycerides	63.67	0.09	0.05	<0.05
Cholesterol	27.30	0.25	0.04	<0.05

ALP, alkaline phosphatase; ALT, alanine aminotransferase; AST, aspartate aminotransferase; BMI, body mass index; GGT, gamma glutamyl transpeptidase.

### Obese patients show increased steatosis compared to controls by multi-echo MRI and Folch value, which is improved after bariatric surgery

**Figure 5:**
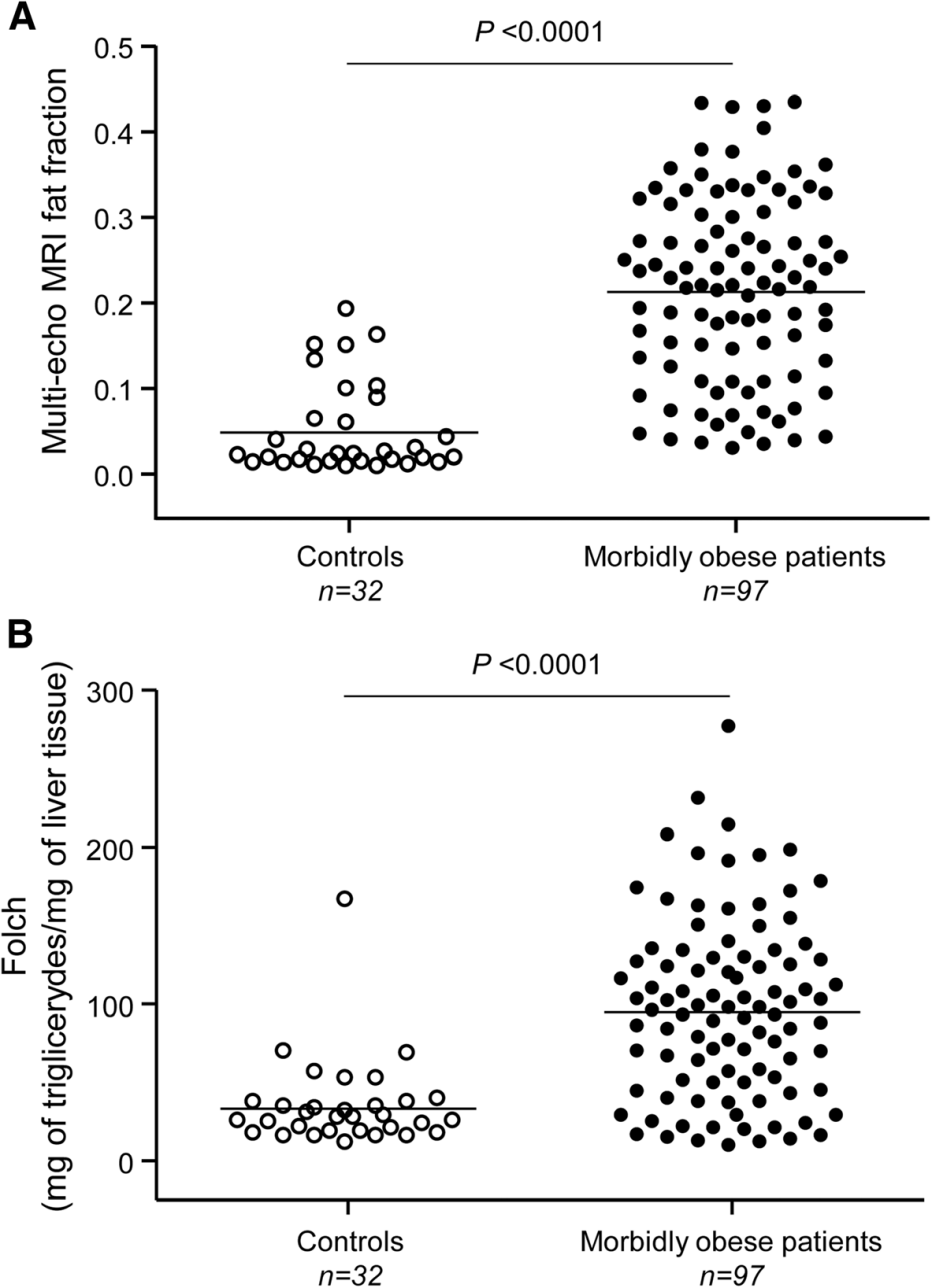
**Obese patients show increased steatosis by multi-echo MRI and Folch value. A)** Multi-echo MRI fat fraction and **B)** Folch value both revealed that obese patients have increased steatosis compared to controls. Mean values are indicated by bars and dots represent the values of each case. MRI, magnetic resonance imaging.

**Figure 6:**
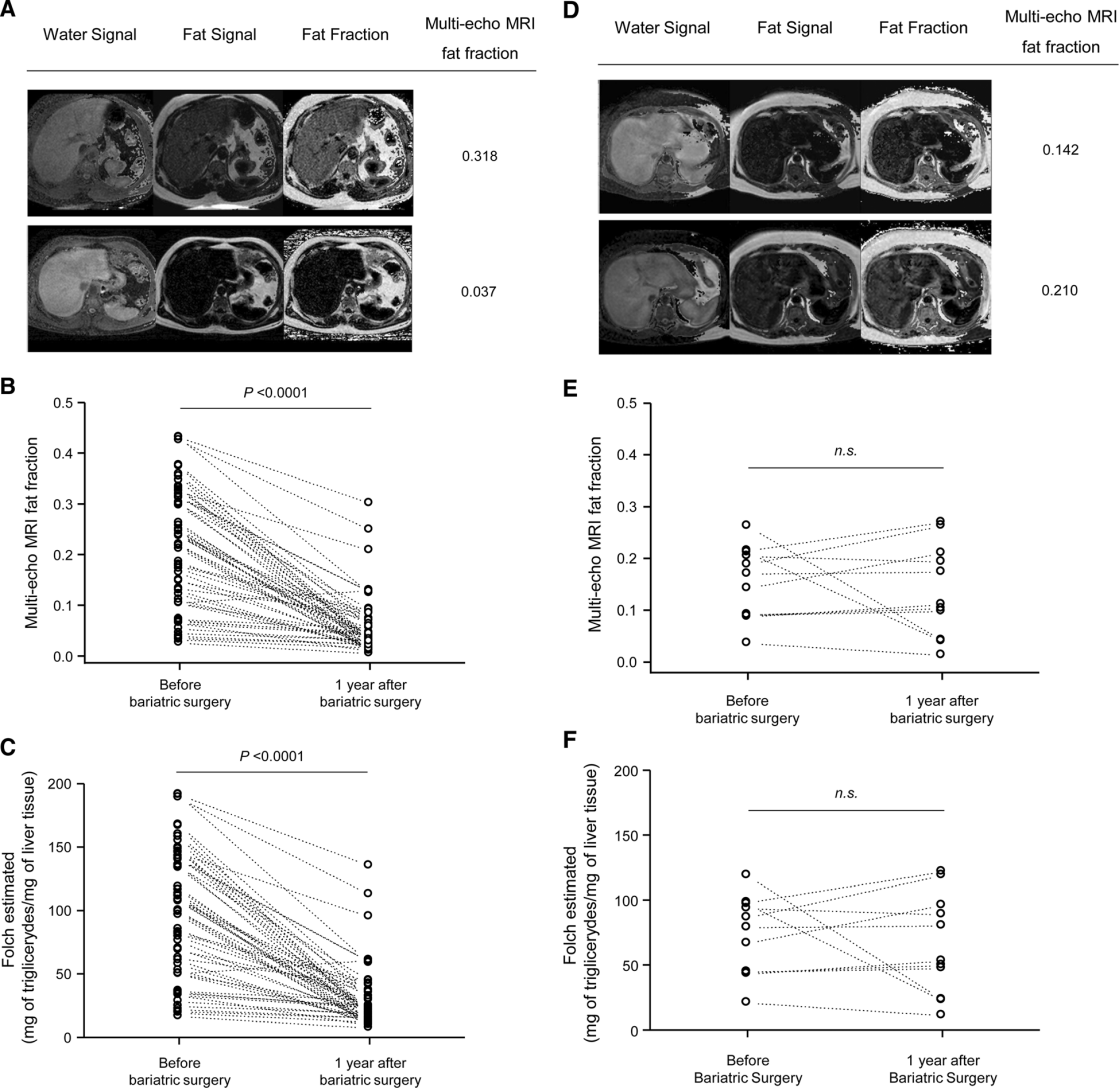
**Bariatric surgery improves steatosis by multi-echo MRI and Folch estimation.** In obese patients who underwent bariatric surgery (n = 56) there was a decrease in the multi-echo MRI fat fraction signal intensities and the Folch value estimated one year after surgery compared to patients who did not receive bariatric surgery (n = 11). **A)** Representative multi-echo MRI images showing reduction of the fat signal and fat fraction in obese patients after bariatric surgery. In obese patients **B)** the multi-echo MRI fat fraction signal intensities and **C)** the Folch estimated one year after surgery were reduced. **D)** Representative multi-echo MRI images showing no changes of the fat intensity and fat fraction in obese patients who did not receive bariatric surgery. In obese patients who did not undergo bariatric surgery **E)** the multi-echo MRI fat fraction signal intensities and **C)** the Folch estimated after one year were not reduced. Dots represent the values of each case. MRI, magnetic resonance imaging.

Finally, from the 97 obese patients, all who had liver resection (n = 11) and those who underwent bariatric surgery at least one year before (n = 56) were monitored with a second multi-echo MRI one year after the operation in order to quantitate steatosis. In this regard, since it is a prospective study, 30 patients did not fulfill the requirement of a year after bariatric surgery and had not undergone the second multi-echo MRI. Obese patients present increased liver steatosis compared to controls using multi-echo MRI fat fraction and Folch value (*P* <0.0001; Figure 5A, B, respectively). However, in patients who underwent bariatric surgery the multi-echo MRI fat fraction signal intensities and the Folch estimated one year after operation were decreased (*P* <0.0001; Figure 6A-C). This effect was not observed in obese patients who did not receive bariatric surgery (Figure 6D-F). Multivariate analysis indicated that the Folch value estimated before bariatric surgery and the weight loss (kg) both improve the Folch estimated one year after surgery (*P* <0.0001; Table 4). Initial weight, gender and age do not correlate with the improvement of steatosis.

**Table 4:** **Final multivariate regression analysis for the MRI-estimated lipid content one year after surgery**

**Steatosis improvement**	**Coefficient**	**Std. Err.**	***P*** **value**
Initial estimated Folch	0.78	0.06	<0.0001
Weight loss	0.68	0.13	<0.0001
Constant	−40.41	7.79	<0.0001

R^2^ = 0.76. MRI, magnetic resonance imaging; Std. Err., standard error.

## Discussion

The key findings reported here indicate that MRI is a non-invasive technique able to quantitate the hepatic triglyceride concentration in NAFLD. Our prospective data from 129 adult patients demonstrated that multi-echo MRI fat fraction strongly correlates (r =0.90/adjusted r^2^ = 0.81) with the biochemical measurement of the hepatic lipid concentration (that is, Folch value), resulting in a novel equation: Folch (mg of triglycerides/g of liver tissue) = 5.082 + (432.104 * multi-echo MRI fat fraction). This formula was afterwards validated in 31 additional adult patients, showing a strong correlation between the Folch measured and MRI-estimated Folch values (r^2^ = 0.98; *P* <0.0001). In addition, multi-echo MRI fat fraction positively correlates with the semi-quantitative measurement of steatosis by histopathological examinations (r = 0.77/adjusted r^2^ = 0.60), but the correlation values were lower than those obtained when the multi-echo MRI fat fraction was correlated with the Folch value. These data, together with the correlation values between Folch and semi-quantitative measurement of steatosis by histopathological examinations (r = 0.71/adjusted r^2^ = 0.50), indicated that the latter is not an accurate method to determinate the hepatic fat content, and the Folch method should be used as an appropriate gold standard for NAFLD. Folch prediction obtained by multi-echo MRI was not improved when other variables were included in the equation. Obese patients show increased steatosis compared to controls using Folch-measured and MRI-estimated values. Bariatric surgery was followed by a decrease in liver fat content as estimated by MRI one year after surgery, and a substantial part of this improvement is explained by the measured Folch value before surgery and the weight loss after surgery. Our data are consistent with the notion that multi-echo MRI is a non-invasive technique that allows the appropriate estimation of the hepatic fat concentration, and can be employed to diagnose and monitor liver steatosis in humans.

Hepatic fat quantification is receiving increasing attention in clinical practice since the prevalence of steatosis associated with obesity is dramatically affecting developed countries [2,3]; moreover, it is also important for liver transplantation since 30% or more fat content contraindicates liver donation [17]. Therefore, it is essential to establish new non-invasive approaches to determine the hepatic fat concentration accurately, allowing the correct diagnosis and monitoring of steatosis. Up to now, there have been no specific biochemical or serological tests able to diagnose the presence of fatty liver, and even less, methods to quantitate the degree of liver steatosis [2]. The standard procedure is the histological estimation of the percentage of hepatocytes containing macrovesicular fat [5], which possesses inherent invasive risks and is subject to the variability of visual estimation between observers [9].

MRI and ^1^H MR spectroscopy (MRS) are currently considered the most promising and sensitive non-invasive methods to assess the total liver fat content [4,18,19], being postulated as potential screening tools for patients at risk of developing NAFLD (that is, with obesity, insulin resistance, type 2 diabetes and/or nutritional disorders) [3,16]. MRI can be performed using gradient-echo chemical shift imaging (Dixon method) as T1-weighted dual echo, triple echo, or multi-echo [19]. However, it is important to take into account that liver iron deposition is able to distort local magnetic fields responsible for T2* shortening, and may ultimately result in signal intensity loss. Given the high prevalence of iron deposition in the liver and the considerable variations of T2* of the liver with consequent errors in the quantification of liver steatosis, T2* bias cannot be considered negligible and must be taken into account. In this regard, multi-echo techniques can easily correct for T2* decay and, thus, should be used to give reliable results [19,20].

Some studies have recently evaluated the diagnostic potential of MRI techniques (that is, two-point Dixon, three-point Dixon, DUAL, Spin echo method and multi-echo gradient-echo) as non-invasive methods to quantitate steatosis. They compared MRI with semi-quantitative analysis of steatosis by histopathological grading, showing different degrees of correlation [1,10,21-23]. It is important to remark that the MRI-biopsy time interval is a key factor in improving the correlation score, since the hepatic fat content may change over time. In this regard, best correlations were obtained with a MRI-biopsy period of seven days or less [10].

Here, 129 adult patients were included in our prospective study. Ninety seven patients were obese, a risk factor for hepatic steatosis, and the other 32 were non-obese patients with potential low grades of steatosis who underwent liver resections for several etiologies but without underlying liver disease. Our data indicate that multi-echo MRI fat fraction positively correlates with the semi-quantitative measurement of steatosis by histopathological examinations. Importantly, the MRI-biopsy period in our study was less than 24 hours, resulting in a robust interpretation of the data from this large cohort of patients. However, histopathological analysis of steatosis is not an adequate gold standard for MRI or ^1^H MRS, since it is a two-dimensional semi-quantitative technique susceptible to inter-individual visual estimation [9]. In this regard, the Folch method is considered the most reliable approach to extract and quantitate lipids [24]. It gives three-dimensional quantitation of the hepatic triglyceride content, thus being an appropriate gold standard [9]. This methodology is not frequently used in clinical practice since it destroys the tissue and, therefore, it cannot be submitted for histological analysis, meaning that important features, such as inflammation, fibrosis or iron deposition, are not evaluated [9]. Our data showed that the histopathological estimation of steatosis positively correlates with the Folch value, although the correlation value was modest, indicating that the semi-quantitative analysis of steatosis presents Folch variability. All of these data indicate that the Folch method is the appropriate three-dimensional approach to determine the hepatic fat concentration and is the correct gold standard for NAFLD.

We and others [16,25-27] have recently reported positive correlations between MRI and Folch in animal models of NAFLD. Thus, we found using experimental (n = 40) and control (n = 10) rat groups that multi-echo MRI strongly correlates with Folch (r^2^ = 0.87) [16]. There are no available studies in the literature, except for a recent pilot study with 18 adult patients who underwent cholecystectomy for symptomatic cholelithiasis [28], that compare the analysis of hepatic steatosis by MRI and the biochemical quantification of triglycerides in human liver biopsies. Our results from a larger cohort of adult patients showed that multi-echo MRI fat fraction strongly correlates with the Folch, resulting in a novel linear regression equation to predict the hepatic triglyceride concentration from multi-echo MRI fat fraction measurements. This formula was afterwards validated in 31 additional patients, showing strong correlation between the measured and estimated Folch values. Thus, obese patients show increased steatosis compared to controls using multi-echo MRI fat fraction and Folch value, and bariatric surgery decreased steatosis in these patients one year after operation. Although the best method to check the accuracy of the developed equation would be the Folch measurement 12 months after surgery, there are ethical problems due to inherent methodological risks. In addition, the MRI-estimated Folch value obtained before bariatric surgery and the weight loss (kg) both positively correlate with the improvement of steatosis one year after operation.

These data strongly suggest that MRI is a valuable diagnostic tool not only to estimate the hepatic lipid concentration, but is also a non-invasive technique to monitor the changes in steatosis occurring in liver tissue. MRI software may automatically generate the fat fraction and, by using our new formula, determine the triglyceride concentration without any inter-observer bias. In addition, multi-echo MRI is a more economic approach than Folch measurement; thus, MRI costs usually are not as much of those resulting from the Folch technique, which include ultrasound-guided liver biopsy and the triglyceride measurement. However, these costs may vary between different hospitals and countries. Overall, this new equation may represent an innovative clinical tool to diagnose and monitor steatosis in patient groups with risk of developing NAFLD, and may also help to discard liver donations of individuals with high fat content.

## Conclusions

Our results identify multi-echo MRI as an appropriate non-invasive approach to predict the hepatic lipid concentration using our novel formula. Since multi-echo software is usually available in MRI units, this may represent an economical non-invasive method to diagnose and monitor steatosis in risk groups with obesity and/or metabolic syndrome.

## Abbreviations

ALP: alkaline phosphatase
ALT: alanine aminotransferase
AST: aspartate aminotransferase
BMI: body mass index
CV: coefficient of variation
FOV: field of view
GGT: gamma glutamyltranspeptidase
MRI: magnetic resonance imaging
MRS: ^1^H magnetic resonance spectroscopy
NAFLD: non-alcoholic fatty liver disease
NASH: non-alcoholic steatohepatitis
ROI: region of interest
Std. Dev: standard deviation
TE: echo time
TR: repetition time
δTE: interval of echo time
